# Carrier of a pathogenic LAMB3 variant: Exploring the interface of genetic skin fragility and cutaneous autoimmunity

**DOI:** 10.1016/j.jdcr.2026.01.034

**Published:** 2026-02-02

**Authors:** Ashley Jakubowicz, Emily R. Nadelmann, Michael A. Occidental, Tobi Klar

**Affiliations:** a State University of New York at Binghamton, Binghamton, New York; b Department of Dermatology, University of Maryland School of Medicine, Baltimore, Maryland; c Department of Dermatology, George Washington School of Medicine and Health Sciences, Washington, District of Columbia; d The Ronald O. Perelman Department of Dermatology, NYU Langone Grossman School of Medicine, New York, New York; e Division of Dermatology, Montefiore Medical Center/Albert Einstein College of Medicine, Bronx, New York

**Keywords:** cutaneous autoimmunity, discoid lupus erythematosus (DLE), junctional epidermolysis bullosa (JEB)

## Case description

A 59-year-old male was referred to dermatology for evaluation of new, persistent, pruritic lesions on the upper back that had developed over several months. He denied any prior history of skin fragility, blistering, or eruptions in that area or elsewhere on the body. There was no history of mucosal involvement, photosensitivity or increased ultraviolet exposure, smoking, medication exposures associated with cutaneous lupus erythematosus, or other known risk factors for cutaneous lupus, and no prior dermatologic diagnoses.

Genetic testing given his family history revealed a heterozygous pathogenic variant in *LAMB3* (c.1903C>T, p.R635∗). His family history was notable for a sibling with a confirmed diagnosis of junctional epidermolysis bullosa (JEB).

**Fig 1:**
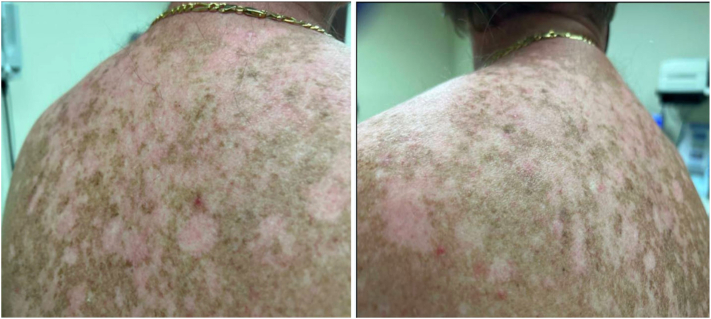
Clinical examination revealed multiple light pink atrophic plaques, some eroded, on upper back.

On examination, the upper back showed several light pink, atrophic plaques, some with overlying scale and central erosion (Fig 1).

**Fig 2:**
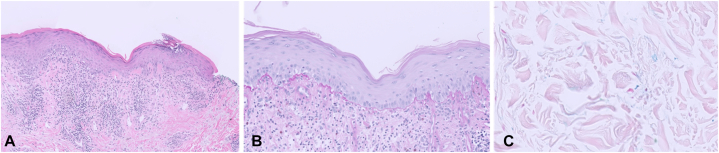
Punch biopsy demonstrated lichenoid lymphocytic inflammatory infiltrate and vacuolar interface changes **(A)**. A PASD stain highlighted basement membrane thickening at the dermo-epidermal junction, **(B)**, and Alcian Blue pH 2.5 stain highlighted mucin between collagen bundles in the dermis **(C)**.

Histopathologic evaluation revealed lichenoid interface dermatitis with basal vacuolar alteration and a band-like lymphocytic infiltrate (Fig 2, *A*). Periodic acid–Schiff with diastase staining showed thickening of the basement membrane zone (Fig 2, *B*), and Alcian Blue (pH 2.5) staining revealed interstitial dermal mucin deposition (Fig 2, *C*). Of note, there was a lack of subepidermal clefting and vesiculation.

Laboratory work-up revealed a positive antinuclear antibody at 1:320 with a speckled pattern and an elevated rheumatoid factor IgM of 8.2 IU/mL. Following evaluation, including rheumatologic assessment, the patient did not meet criteria for systemic lupus erythematosus and had no evidence of systemic involvement.

## Question 1: What is the diagnosis?

**A.** Junctional epidermolysis bullosa
**B.** Discoid lupus erythematosus
**C.** Subacute cutaneous lupus erythematosus
**D.** Granuloma annulare
**E.** Polymorphic light eruption

## Answer

**B.** Discoid lupus erythematosus – Correct.

## Discussion

JEB is a rare inherited blistering disorder caused by biallelic mutations in genes encoding components of the dermoepidermal basement membrane, most commonly *LAMB3*.^1^, ^3^^,^^1^, ^2^ While carriers of pathogenic *LAMB3* variants are typically asymptomatic, emerging evidence suggests that heterozygous mutations may have subclinical or modulatory effects on skin integrity, wound healing, or immune response.^3^

This case is, to our knowledge, the first report of discoid lupus erythematosus (DLE) arising in a patient who is a carrier of a pathogenic *LAMB3* variant, in the absence of clinical or histologic evidence of JEB. The patient had no prior history of skin fragility, blistering, or eruptions at the site of DLE involvement, raising the possibility of a unique interaction between genetic susceptibility and autoimmune skin disease.

This case raises important questions about the potential for genetic variations in *LAMB3* to interact with environmental triggers, such as ultraviolet radiation, in the development of autoimmune skin conditions. Heterozygosity for a pathogenic variant in a gene associated with JEB may not only impact skin integrity but also subtly influence immune responses to endogenous or environmental stimuli.

The development of DLE at a site with no previous skin trauma or scarring distinguishes this case from those of secondary autoimmune processes that typically arise in chronically injured skin. These findings suggest that haploinsufficiency of *LAMB3* may subtly compromise basement membrane integrity or immune surveillance in the skin, potentially increasing susceptibility to autoimmunity even in the absence of overt blistering.

## Conflicts of interest

None disclosed.

## References

[1] Pfendner E.G., Lucky A.W., MP Adam, D Bick, GM Mirzaa (2018). GeneReviews [Internet].

[2] MedlinePlus Genetics. LAMB3 gene: laminin subunit beta 3. U.S. National Library of Medicine. MedlinePlus genetics website. 2019. Accessed December 1, 2025. https://medlineplus.gov/genetics/gene/lamb3/#resources

[3] Wen D., Hunjan M., Bardhan A. (2024). Genotype-phenotype correlation in junctional epidermolysis bullosa: signposts to severity. J Invest Dermatol.

